# The recent emergence of a highly related virulent *Clostridium difficile* clade with unique characteristics

**DOI:** 10.1016/j.cmi.2019.09.004

**Published:** 2020-04

**Authors:** H.A. Shaw, M.D. Preston, K.E.W. Vendrik, M.D. Cairns, H.P. Browne, R.A. Stabler, M.J.T. Crobach, J. Corver, H. Pituch, A. Ingebretsen, M. Pirmohamed, A. Faulds-Pain, E. Valiente, T.D. Lawley, N.F. Fairweather, E.J. Kuijper, B.W. Wren

**Affiliations:** 1) Department of Pathogen Molecular Biology, London School of Hygiene and Tropical Medicine, London, UK; 2) Division of Bacteriology, National Institute for Biological Standards and Controls, South Mimms, Potters Bar, UK; 3) Analytical Biological Service Division, National Institute for Biological Standards and Controls, Potters Bar, UK; 4) National Reference Laboratory for CDI Surveillance, Department of Medical Microbiology and RIVM, Leiden University Medical Centre, Leiden, the Netherlands; 5) Public Health Laboratory London, Division of Infection, The Royal London Hospital, London, UK; 6) Wellcome Sanger Institute, Wellcome Genome Campus, Hinxton, UK; 7) Department of Medical Microbiology, Medical University of Warsaw, Warsaw, Poland; 8) Department of Microbiology, Oslo University Hospital, Oslo, Norway; 9) Department of Infection Prevention, Oslo University Hospital, Oslo, Norway; 10) Department of Molecular and Clinical Pharmacology, The University of Liverpool, Liverpool, UK; 11) CMBI, Imperial College London, London, UK

**Keywords:** *Clostridium difficile*, *Clostridium difficile* infection, Next-generation sequencing, RT023, Trehalose, S-layer, Clade 3

## Abstract

**Objectives:**

*Clostridium difficile* is a major global human pathogen divided into five clades, of which clade 3 is the least characterized and consists predominantly of PCR ribotype (RT) 023 strains. Our aim was to analyse and characterize this clade.

**Methods:**

In this cohort study the clinical presentation of *C. difficile* RT023 infections was analysed in comparison with known ‘hypervirulent’ and non-hypervirulent strains, using data from the Netherlands national *C. difficile* surveillance programme. European RT023 strains of diverse origin were collected and whole-genome sequenced to determine the genetic similarity between isolates. Distinctive features were investigated and characterized.

**Results:**

Clinical presentation of *C. difficile* RT023 infections show severe infections akin to those seen with ‘hypervirulent’ strains from clades 2 (RT027) and 5 (RT078) (35%, 29% and 27% severe CDI, respectively), particularly with significantly more bloody diarrhoea than RT078 and non-hypervirulent strains (RT023 8%, other RTs 4%, p 0.036). The full genome sequence of strain CD305 is presented as a robust reference. Phylogenetic comparison of CD305 and a further 79 previously uncharacterized European RT023 strains of diverse origin revealed minor genetic divergence with >99.8% pairwise identity between strains. Analyses revealed distinctive features among clade 3 strains, including conserved pathogenicity locus, binary toxin and phage insertion toxin genotypes, glycosylation of S-layer proteins, presence of the RT078 four-gene trehalose cluster and an esculinase-negative genotype.

**Conclusions:**

Given their recent emergence, virulence and genomic characteristics, the surveillance of clade 3 strains should be more highly prioritized.

## Introduction

*Clostridium difficile* remains a major global pathogen; disease severity and relapse incidence have not abated, and community-acquired infections have increased [1]. It can be divided into five clades of virulent strains [2]. The most understudied is clade 3, dominated by PCR ribotype (RT) 023 strains [2]. RT023 has been reported primarily in Europe [3] and is among the top ten most common *C. difficile* PCR ribotypes in England [4] (CDRN report 2013–2015) and the Netherlands (unpublished data of the Dutch *C. difficile* Reference Laboratory). RT023 infections are not associated with increased mortality despite causing a high level of deleterious biomarkers (e.g. neutrophil counts) in patients and having toxin profiles similar to clade 2 (RT027) and clade 5 (RT078) strains [5,6]. However, disease severity with RT023 has been reported as similar to ‘hypervirulent strains’, particularly in elderly individuals [7], and is frequently associated with a relapse of *C. difficile* infection (CDI) [3].

This study investigates the clinical presentation and phylogeny of *C. difficile* clade 3, uncovering and characterizing unique features of these strains.

## Methods

### Clinical data collection and analysis

A cohort study was performed. Clinical data from the Dutch national CDI sentinel surveillance from May 2009 until February 2018 were used to analyse the clinical characteristics of CDI episodes due to RT023. For this sentinel surveillance, all hospitalized patients >2 years old, with clinical signs or symptoms of CDI in combination with a positive test for *C. difficile* toxins or toxigenic *C. difficile*, in Dutch participating hospitals, were registered. The indication for testing for CDI and the assay or algorithm that is used to diagnose CDI is chosen by the local laboratory.

Using classification criteria based on expert opinion that were previously used [8], CDI is classified as severe if one or more of the following conditions was present; fever (temperature of 38°C or higher) and leucocytosis (>15 × 10^9^/L), diarrhoea with hypoalbuminaemia (<20 g/L) and/or dehydration, pseudomembranous colitis and/or bloody diarrhoea. A complicated course is defined as the need for surgical procedure, admission to intensive care unit and/or mortality (CDI-related or non-CDI-related) within 30 days after CDI diagnosis [8].

Our primary aim was to test the null hypothesis that RT023 causes the same proportion of severe CDI as non-hypervirulent ribotypes. Therefore, clinical characteristics and 30-day outcome of CDI episodes due to RT023 were compared with CDI episodes due to other ribotypes (excluding hypervirulent strains RT027 and RT078/126). Thereafter, the results of the RT023 group were compared with the results of four pre-specified groups; RT027 and RT078/126, which are well-known hypervirulent strains, and RT001 and RT014/020/295, which are non-hypervirulent strains that are common in the Netherlands. Each time, the results of the RT023 group were compared with the results of one other group. Some ribotypes were merged into one group because they are hard to distinguish with PCR ribotyping. Further details are given in the Supplementary material (Appendix S1).

Data are presented as number of cases (percentage) or percentage (95% confidence interval). Age is presented as median (first quartile, third quartile), because of the skewed distribution. Categorical variables were compared by a Pearson's chi-square test or Fisher's exact test for expected frequencies <5, and numerical variables were compared by a Wilcoxon rank-sum test. To identify the effect of RT023 on CDI severity, a multivariable logistic regression analysis was performed with age and sex as covariates. A p value of <0.05 was considered statistically significant. STATA SE version 12.1 statistical software (StataCorp, College Station, TX, USA) was used for statistical analysis.

### Ethics

This was an observational study, using data that are already collected in the Dutch national CDI surveillance. This national surveillance programme has existed since 2009 and collects microbiological and clinical data from all hospitalized patients with CDI in the participating hospitals in the Netherlands. The surveillance has been developed by our National Institute of Public Health. There were no additional data or isolates/materials specifically for this study collected and no actions were requested from patients.

### Whole-genome sequencing

CD305 genomic DNA was sequenced using 454 pyrosequencing (GS-FLX pyrosequencing) to generate 3-kb paired-end libraries and Illumina GAII paired-end libraries of 400-bp insert size and 108-bp read length. The resulting sequence was assembled using Newbler and Velvet and the assemblies were combined using Newbler [9,10]. Identification and annotation of coding sequences (CDS) were generated using PROKKA [11] with a bespoke *C. difficile* library. The assembled and annotated genome is available at ERS2502454. For 79 study isolates, genomic DNA libraries were created using a Nextera XT kit (Illumina, San Diego, CA, USA) and data were obtained using the MiSeq sequencing system (Illumina).

### Whole-genome bioinformatics analysis

The sequence data were processed according to a standard protocol as previously described [12] (see Supplementary material, Appendix S1). Single nucleotide polymorphism (SNP) loci were identified with a samtools Q-score ≥30, coverage ≥10 and 80% of contributing reads. Pipeline, phylogenetic and post-analyses were carried out using Perl, R and RAxML [13].

### Glycoprotein detection

Glycosylated proteins were detected using a Pierce Glycoprotein Staining Kit according to the manufacturer's instructions (Pierce Biotechnology, Rockford, IL, USA) (see Supplementary material, Appendix S1).

## Results

### CDI in hospitalized patients due to RT023 strains is severe comparable with RT027 and RT078 strains

Between May 2009 and February 2018, 5359 samples from hospitalized patients in 24 hospitals in the Netherlands were PCR-ribotyped within the context of the national *C. difficile* surveillance programme. Clinical data were complete in 4387 cases. RT023 accounted for 141 cases of CDI, a mean proportion of 2.4% (95% CI 2.0%–2.8%), which remained consistent within the study period.

Demographic data, clinical characteristics and 30-day outcome of patients with CDI due to RT023 were compared with data of five other pre-specified ribotype groups, shown in Table 1. There were no significant differences in age and sex between the RT023 group and the other groups, except for higher age in the RT001 group.

**Table 1:** Comparison of clinical characteristics of patients with RT023 versus other ribotypes (excluding RT027 and RT078/126), RT027, RT078/126, RT014/020/295 and RT001

	RT023 (*n* = 141)	Primary outcome	Hypervirulent strains		Non-hypervirulent strains		All info available
		Others (*n* = 4368)	RT027 (*n* = 116)	RT078/126 (*n* = 734)	RT014/020/295 (*n* = 962)	RT001 (*n* = 699)	
Age	71.4 (10.0–97.7)	71.3 (1.9–102.3)	73.2 (11.2–91.5)	70.9 (5.2–100.7)	70.4 (2.1–99.2)	76.0 [3.3, 96.7]*	5359/5359
Men	71 (50)	2095 (48)	63 (54)	365 (50)	444 (46)	344 (49)	5356/5359
Severe CDI	45 (35)	880 (22)*	30 (29)	188 (27)	185 (21)*	104 (16)*	4948/5359
Dehydration and/or hypoalbuminaemia	25 (20)	450 (11)*	14 (14)	100 (15)	97 (11)*	44 (7)*	4940/5359
Bloody diarrhoea	10 (8)	192 (5)	6 (6)	25 (4)*	34 (4)*	24 (4)*	4948/5359
Pseudomembranous colitis	8 (6)	159 (4)	6 (6)	41 (6)	28 (3)	21 (3)	4948/5359
Fever and leucocytosis	11 (9)	295 (7)	9 (9)	76 (11)	64 (7)	36 (6)	4940/5359
Complicated course	13 (12)	485 (14)	21 (23)*	104 (17)	78 (10)	95 (17)	4387/5359
Overall mortality	10 (9)	428 (12)	18 (19)*	98 (16)*	68 (9)	86 (15)	4387/5359
CDI mortality	2 (2)	104 (3)	4 (4)	29 (5)	16 (2)	27 (5)	4387/5359
Community onset	75 (54)	1545 (36)*	31 (27)*	272 (37)*	356 (38)*	155 (23)*	5283/5359
CDI last 8 weeks	22 (27)	684 (25)	12 (20)	133 (29)	161 (27)	115 (25)	3312/5359

Abbreviations: CDI, *Clostridium difficile* infection; HCF, health-care facility; LTCF, long-term-care facility; RT, ribotype.

Data are presented as number of cases (percentage). Age is presented as median (first quartile, third quartile), because of the skewed distribution. Categorical variables were compared by a Pearson's chi-square test and numerical variables were compared by a Wilcoxon rank-sum test. An asterisk (*) represents a p < 0.05, when comparing with RT023.

The primary question was whether CDI due to RT023 was more severe when compared with all non-hypervirulent ribotypes, which was confirmed by our results (p 0.000: 35% (27%–44%) versus 22% (21%–23%)), also after correcting for sex and age. No significant differences of severity were found when RT023 was compared with ‘hypervirulent’ strains RT027 and RT078/126 (p 0.310 and p 0.065, respectively, RT023: 35% (27%–44%), RT027: 29% (20%–38%), RT078/126: 27% (24%–31%)), also not after correction for sex and age. Bloody diarrhoea was more frequently reported in RT023 infections compared with RT078/126 infections (p 0.031), RT014/020/295 (p 0.036) and RT001 (p 0.037) (RT023: 8% (3%–13%), RT078/126, 4% (2%–5%), RT014/020/295 4% (3%–5%), RT001 4% (2%–5%)). When compared with non-hypervirulent RT001 and RT014/020/295 isolates, with or without correcting for sex and age, RT023 presented with significantly more severe symptoms (p 0.000 for both, RT023: 35% (27%–44%), RT001: 16% (13%–19%), RT014/020/295: 21% (18%–23%)), such as more frequent diarrhoea with dehydration and/or hypoalbuminaemia. However, the outcomes of CDI due to RT023 in terms of a complicated course, including mortality, were comparable with outcomes of CDI due to RT001, RT014/020/295 and all non-hypervirulent ribotypes. RT027 and RT078/126 infections showed higher overall mortality than RT023 (p 0.032, p 0.049, respectively, RT023: 9% (4%–14%), RT027: 19% (11%–27%), RT078/126: 16% (13%–19%)) but CDI-attributable mortality was similar between these groups (p 0.415, p 0.206, respectively, RT023: 2% (0–6%), RT027: 4% (1–11%), RT078/126: 5% (3%–7%)). There were significantly more complicated courses in patients with CDI due to RT027 compared with RT023 (p 0.038, 23% (14%–31%) versus 12% (6%–18%) respectively), but no significant differences were observed between RT078/126 and RT023 (p 0.144, RT078/126 17% (14%–20%)).

Comparison of RT023 with all groups in this study revealed that the onset of symptoms of CDI due to RT023 was more frequently at home and less often in healthcare facilities (p 0.000 compared with all other groups). Subgroup analysis of community-onset and hospital-onset CDI can be found in the Supplementary material (Table S1). The number of episodes that were recurrences of a previous CDI episode 2–8 weeks earlier was the same in RT023 episodes compared with all other groups (Table 1).

### Clade 3 strains are highly related

**Fig. 1:**
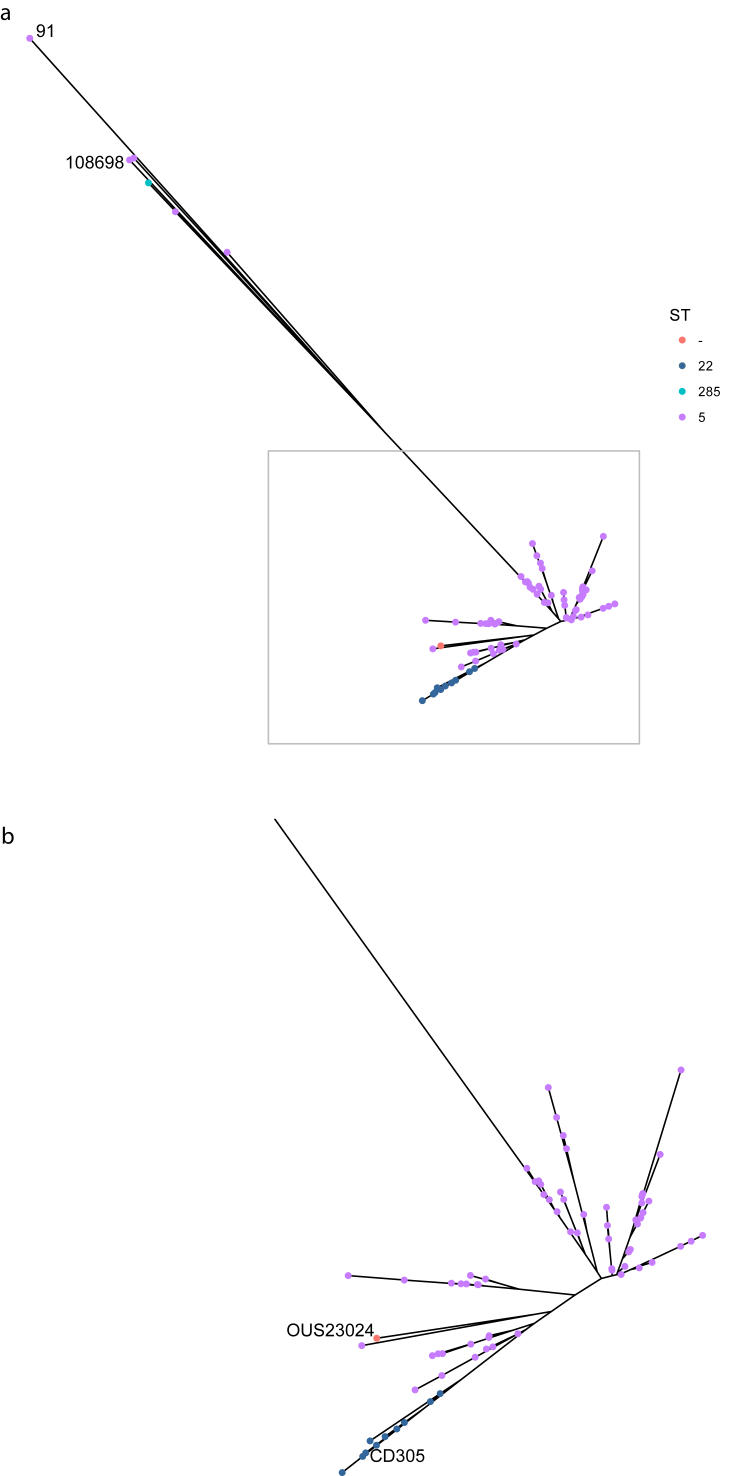
Phylogenetic tree by multilocus-sequence typing (MLST). Phylogenetic tree of 86 strains generated from analysis of high-quality single nucleotide polymorphisms (SNPs) and coloured by MLST. (a) Full tree, with two cohort outliers (samples 91 and 108676), Ox2183 and three Chinese strains. (b) The large, temporally indistinguishable main cluster, with reference CD305 and novel MLST strain OUS23024 indicated.

A high-quality [14] draft genome of strain CD305 (RT023) was generated and is presented here as a robust reference for this lineage. Further strains were sourced from across Europe (see Supplementary material, Table S2), with this study comprising 86 strains: CD305 (reference); 79 (out of 170 strains with the whole-genome sequence (WGS) available); and six published clade 3 strains [15,16] (see Supplementary material, Table S3), the largest RT023 genomic collection. Multilocus sequence types (MLST) were identified *in silico* from *de novo* assemblies*.* The six published strains matched their published MLST with new strains composed of 68 ST005, ten ST022 and one novel sequence type (strain OUS23024) (Fig. 1).

The 79 core strains were aligned to the CD305 reference strain and a set of 19 262 (<0.5% of the 4.2-Mbp genome) high-quality SNP loci was identified. The individual strains were very closely related with only between 58 and 7876 pairwise SNP differences, with a mean of 1767 SNPs (mean 9.2% of 19 262 SNPs; maximum 40.9%) equating to >99.8% pairwise identity between strains. A phylogeny was created from the SNPs of all 86 strains that reinforces the conclusion of little genetic diversity within clade 3 strains (Fig. 1). From our 80 strains there were two outliers: strains 91 and 108698, which are not RT023 (Fig. 1a, see Supplementary material Appendix S1 and Fig. S3). The unassigned MLST strain (OUS23024) diverged slightly from the main population (Fig. 1b). No significant relationship was found with any phenotypes, including the infection date (2007–2014) or geographic origin (see Supplementary material, Table S2 and Fig. S1). Details on MLST and ribotype divergence can be found in the Supplementary material (Appendix S1).

There is high conservation in all 86 strains of larger clade-specific genetic features such as the pathogenicity locus (PaLoc), binary toxin C. difficile toxin (CDT), PaLoc phage insertion and type B flagella glycosylation cluster (see Supplementary material, Tables S3 and S4). The only common antibiotic resistance marker is *gyrB* (V426D) related to fluoroquinolone resistance. Analysis of 12 Polish RT023 strains for fluoroquinolone resistance revealed resistance to ciprofloxacin but sensitivity to moxifloxacin (see Supplementary material, Table S5).

### A unique trehalose metabolism genotype is present in clade 3 strains

**Fig. 2:**
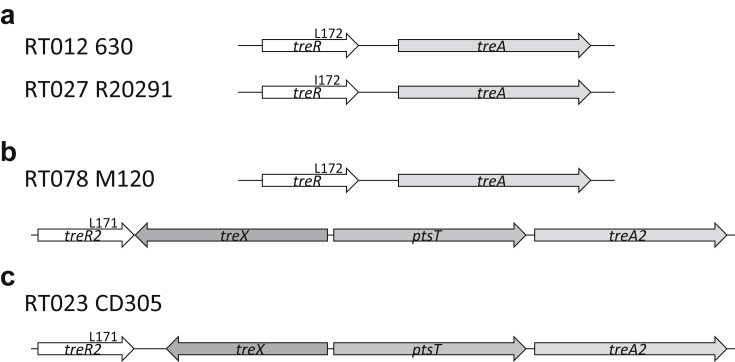
Clade 3 show a unique trehalose genotype. Schematic demonstrating the three trehalose metabolism genotypes observed in *Clostridium difficile* with clade 3 strains lacking the primary trehalose metabolism cluster. (a) RT012 630 and RT027 R20291 genotypes of a primary trehalose cluster, with the L172I single nucleotide polymorphism associated with increased metabolism of trehalose. (b) RT078 M120 genotype with primary and secondary trehalose metabolism gene clusters observed. (c) RT023 CD305 trehalose genotype with only the secondary cluster including a truncated *treX* gene.

Analysis of clade 3 strains for two trehalose clusters described as being important in global dissemination and virulence of *C. difficile* [17] showed a trehalose genotype unique to these strains. The primary cluster, in which SNP L172I defines increased metabolism in RT027 (clade 2) (Fig. 2a), was absent from all clade 3 genomes analysed. This coincides with polymorphisms and a large deletion in sugar metabolism genes in clade 3, including β-glucosidase genes (see Supplementary material, Appendix S1 and Fig. S4). However, the RT078 (clade 5) second cluster (Fig. 2b) was observed in all strains. Polymorphisms exist between the RT078 cluster in M120 cluster and RT023 CD305, with the most significant difference being a truncation of *treX* (Fig. 2c). Between clade 3 strains there are only a small number of SNPs, predominantly in strain 91 (see Supplementary material, Appendix S1).

### Clade 3 have a glycosylated surface

**Fig. 3:**
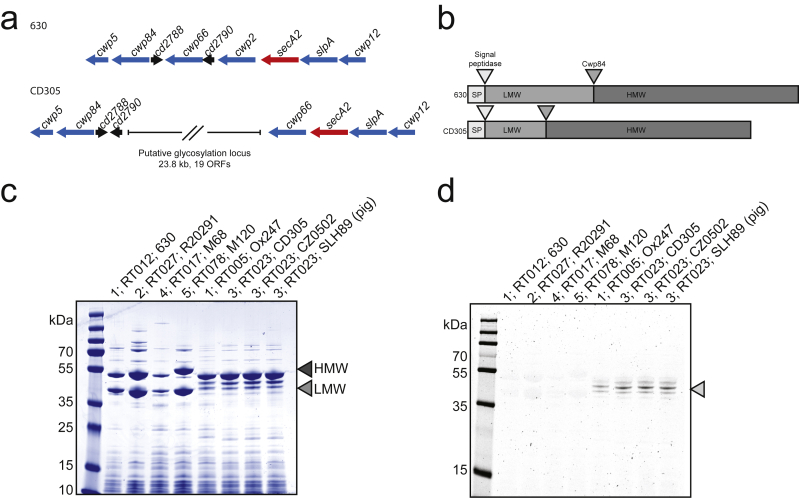
Insertion of a glycosylation cluster results in S-layer glycosylation. RT023 contains a glycosylation cluster within the *slp* gene island. (a) Genomic organization of the *slp* gene island in 630 (Clade 1) and CD305 (Clade 3) showing loss of Cwp2 and acquisition of a gene cluster comprising putative glycosylation genes (adapted from Kirk et al. [18]). (b) Structure of SlpA in 630 and CD305 showing Cwp84 cleavage sites and truncated low-molecular weight (light grey) in CD305. (c) Coomassie staining of S-layer protein preparations from representative strains from each clade showing characteristic double banding for high-molecular weight and low-molecular weight S-layer protein (grey arrows). (d) Periodic acid–Schiff staining of glycans in S-layer preparations.

SlpA is the major surface protein of *C. difficile* comprised of high- and low-molecular-weight proteins (HMW and LMW S-layer protein (SLP)) [18]. A putative glycosylation cluster within the *slp* gene island (Fig. 3a) for S-layer cassette type 11, SLCT11 [18], has been previously reported [19]. and 83 of the 86 strains contain this feature (see Supplementary material, Table S3 and Fig. S2). Strains 91, Ox2183 and WCHCD103, from which this feature is absent, are genetically distinct from other strains within this clade, with alternative *slpA* genes. In RT023 the *slpA* gene encodes a smaller LMW SLP than in other clades, predicted at approximately 18 kDa (Fig. 3b). S-layer extracts of representative strains from each of the five clades of *C. difficile* show two distinct bands of equimolar ratio representing the HMW and LMW SLPs in clades 1, 2, 4 and 5 by Coomassie brilliant blue staining (Fig. 3c). Strain Ox247 (RT005, clade 1) containing SLCT11 [20] along with S-layer preparations from three representative RT023 strains show an alternative pattern of SLPs. HMW SLP migrates at its expected molecular weight, but a band at 18 kDa for LMW SLP is absent. A periodic acid–Schiff assay to stain for glycans on S-layer preparations showed glycosylated proteins at ~45 kDa only in strains containing the glycosylation cluster, demonstrating the presumed functionality of the cluster and glycosylation of S-layer proteins.

## Discussion

This study provides a comprehensive analysis of clade 3 strains of *C. difficile* with an extensive report of RT023 CDI and detailed WGS analysis. The clinical characteristics of hospitalized patients with CDI due to RT023 showed CDI severity similar to the ‘hypervirulent’ RT027 and RT078/126, with comparable CDI-related mortality, although overall mortality was lower in RT023 as previously reported [6]. The phylogeny of clade 3 strains is compact, barring six distinct outliers. In contrast to clade 2 strains (RT027), clade 3 strains show great similarity consistent with a recently emerged clade under little selective pressure to evolve [21]. WGS analysis revealed a unique trehalose genotype and conserved incorporation of a glycosylation cassette into the clade 3 genomes, which was shown to glycosylate the S-layer.

Considering previous investigations, the severity of disease is probably due to the production of binary toxin and the TcdC stop codon in RT023 [5]. Recurrent infections due to RT023 were similar to other ribotypes. This contrasts with an earlier study, where RT023 was dominant among recurrent infections [22]. We also observed more community acquisition of RT023 symptoms, but current reports cannot explain this observation. Circulating strains are unlikely to be the source of RT023 with no representation of RT023 in a small group of *C. difficile* carriers [23] and a low representation in *C. difficile* infections in the community [24]. The low proportion (2.4%) of CDI due to RT023 observed in this study in the Netherlands is consistent with a previous study on CDI in Europe [3].

Strengths of this study are the high sample size, multicentre design with high number of hospitals in different geographic regions, and 10 years of available data, making the data generalizable for hospitalized patients. Similarly, a sample size of over 80 strains across 8 years from a variety of pan-European sources for WGS, as well as published strains including Chinese strains, enabled us to understand the phylogeny of clade 3 in much greater detail. Limitations of the clinical data include the location of symptoms onset being documented but not the location of *C. difficile* acquisition. Furthermore, no data were available regarding co-morbidity, which might affect the outcome. Regarding severity of disease, occasionally not all laboratory parameters needing laboratory results were measured and included.

It has recently been shown that S-layer glycosylation is important for adherence to Caco-2 intestinal epithelial cells but not for biofilm formation [20]. Therefore, glycosylation of the S-layer in clade 3 may be important for colonization but not persistence, explaining a low level of carriage and recurrence of these strains. Despite severe clinical presentation this clade is not as widely disseminated as other clades. The emergence of RT027 and RT078 strains has been linked to an increased ability to metabolise the food additive trehalose [17]. RT023 strains contain the second four-gene cluster, corroborated by a recent study of trehalose genes in all clades of *C. difficile*. The presence of only the secondary cluster and the SNPs between RT023 and RT078 may result in a difference in uptake and metabolism of trehalose between these strains, which could explain the relatively reduced prevalence of RT023 strains compared with RT078 and RT027 strains globally. No link between trehalose and adverse disease outcomes has been suggested [25]. Meanwhile, the emergence of epidemic clade 2 strains has also been linked to environmental spore contamination and the acquisition of fluoroquinolone resistance, which is less pronounced for clade 3 strains [21]. More analysis on sporulation in clade 3 is required because reduced sporulation efficiency and survival outside the human host have been reported [26]; however, a recent study highlighted a clade 3 strain in China that had high sporulation and germination rates [27].

It remains to be determined why evolutionarily distinct clades of *C. difficile* are emerging simultaneously to cause disease in human populations, or if *C. difficile* is evolving into subspecies [28]. Our study suggests that a heightened awareness and continued surveillance of RT023 strains globally should be a current imperative.

## Data availability

Sequence data that support the findings of this study have been deposited in EMBL Nucleotide Sequence Database with accession code PRJEB26893 and CD305 reference genome ERS2502454.

## Transparency Declaration

The authors declare no conflicts of interest. The work was supported by The Wellcome Trust (Grant Reference 102979/Z/13/Z and 098051) and the Medical Research Council (Grant Reference MR/K000551/1).

## Author contributions

Concept and design of study: HAS, MDC and BWW. Genomic assembly and annotation: MDP, HPB and RAS. Genomic analysis: HAS and MDP. Phenotypic experiments: HAS. Fluoroquinolone testing: HP. Clinical analysis: KEWV, MJTC and EJK. The manuscript was drafted by HAS, MDP, KEWV and BWW, and revised by all authors.

## Appendix A Supplementary data

Multimedia component 1 Clade 3 strains sourced for the study. Multimedia component 1

Multimedia component 2 Summary of multilocus sequence typing and genomic features of 86 clade 3 strains. Multimedia component 2

Multimedia component 3 Single nucleotide polymorphism analysis of clade 3 PaLoc and CDT regions. Multimedia component 3

Multimedia component 4 Fluoroquinolone resistance in Polish RT023 strains. Multimedia component 4
