# Supplementary material for: The recent emergence of a highly related virulent Clostridium difficile clade with unique characteristics
Source: Clin Microbiol Infect. 2020 Apr;26(4):492–8. doi: 10.1016/j.cmi.2019.09.004 (PMC7167513; doi:10.1016/j.cmi.2019.09.004)
Supplement: Multimedia component 5 [file mmc5.docx]

**Supplementary Material**

**Appendix S1 Supplementary Text**

**Supplementary methods**

**Clinical data collection and analysis**

For the clinical data collection, *C. difficile* isolates and clinical data of the National Dutch CDI surveillance were used. The National Dutch CDI surveillance started in May 2009. Twenty-four Dutch hospitals participate in this surveillance. They send all *C. difficile* isolates or stool samples to the National Reference laboratory in Leiden University Medical Centre for PCR-ribotyping. PCR ribotyping is performed using the MultiNA and/or capillary gel electrophoresis.

Using data of electronic medical records, local microbiologists or infection control practitioners fill in online questionnaires with questions on clinical and demographic information of the patients with CDI that meet with the case definition and do not meet with the exclusion criteria.

An outbreak is defined as >2 isolates of the same type detected less than 7 days apart in one hospital, either with onset of symptoms on the same ward or accompanied by an increased CDI monthly incidence within the hospital (1). Epidemiologically and genetically related cases, not fulfilling the criteria for an outbreak are called ‘clusters’.

The CDI case definition that is used for the Dutch CDI surveillance:

Every sample of a patient with CDI that is admitted to a hospital and that meets with the following criteria: a patient of at least 2 years old with clinically diagnosed diarrhea or toxic megacolon, and a positive test for the presence of *C. difficile* toxins in feces or detection of a toxin producing *C. difficile* strain via culture/PCR or pseudomembranous colitis found during endoscopy/surgery or histopathology.

Inclusion criteria for the national CDI surveillance:

- Patients that are admitted to a participating hospital with suspicion of CDI and that have a positive *C. difficile* test on feces as described in the case definition.

Exclusion criteria for the national CDI surveillance:

- Every sample of a patient that is younger than 2 years old.
- Every additional positive *C. difficile* sample of the same patient within 2 weeks of a previous sample.
- Every sample of the same episode of diarrhea.
- Samples of persons that are not admitted to the hospital (e.g. daycare, nursery home, outpatients).

Definitions:

- Diarrhea is defined as watery stools more than 3 times per 24 hours for at least 2 days and a defecation frequency that is higher than what is normal for the patient.
- Toxic megacolon is defined as radiologic signs of swelling of the colon and signs of a severe systemic inflammatory response.

Missing data were listed in a table. Since missing data in this setting was not expected to be a confounding factor, no corrections were made for missing values. Mostly, persons that filled in the clinical data and outcomes were not involved in the treatment of the patient. Reasons for missing clinical data could be communication problems in the laboratories or oblivion.

**Bacterial study isolates and growth conditions**

Study isolates are shown in Supplementary Table S3. *C. difficile* cultures were cultured anaerobically (Don Whitley Scientific, West Yorkshire, United Kingdom) at 37°C in BHIS broth (BHI broth (Oxoid) supplemented with 0.1 % L-cysteine (Sigma), 0.5 % tryptose (Bacto), and 1.5 % agar for BHIS agar plates (Bacto).

**DNA extractions**

CD305 genomic DNA was extracted by phenol-chloroform. For 79 study isolates genomic DNA (gDNA) was extracted from 5 ml overnight cultures. Cells were harvested at 4000 x *g* 10 min, resuspended in 200 μl 0.2 M glycine pH 2.2 and incubated at room temperature 20 min with rotation to remove surface proteins and polysaccharides. Cells were harvested at 17,000 x *g* 10 min and the supernatant discarded. Cell pellets were resuspended in 200 μl nuclease free H_2_O with 1.5 mg/ml RNaseA, transferred to 0.1 mm zirconian beads, and lysed with 1 ml CLS-TC (MP Bio) by Ribolyser for 40 s. Suspensions were incubated at 37°C for 1 hour and then processed with FastDNA Spin kit (MP Bio) and DNA eluted in 100 μl ultra-pure H­_2_O.

**Genome assembly**

The CD305 genome was manual edited with GAP4 and orientation against the 454 scaffolds using *C. difficile* R20291 as a comparator allowed computational sequence gap closure using IMAGE and PCR and capillary sequencing (2, 3). iCORN was used to identify and correct sequence errors (<http://icorn.sourceforge.net/>). The PaLoc region in CD305 was further validated using capillary sequencing across the entire region. The sequence data were processed as follows: raw FASTQ files had sequence adapters removed and trimmed using Trimmomatic (4). The trimmed reads were aligned to the CD305 reference (accession number: ERS2502454) using bwa (5). Alignments were transformed and assessed using picard ([**http://broadinstitute.github.io/picard**](http://broadinstitute.github.io/picard)), samtools (6) and vcftools (7). Over 98% of post-trimmed reads aligned (mean: 98.1%; range 92.3% to 99.9%). Additionally, the reads were assembled using Velvet (8) and VelvetOptimiser (https://github.com/tseemann/VelvetOptimiser).

**Cell fractionation**

For preparation of S-layer proteins, cultures were harvested at 4,000 x *g* 10 min and resuspended to OD 20/ml in glycine (pH 2.0). Cells were incubated for 20 min RT with rotation before being harvested at 14,000 x *g* 10 min 4°C. Supernatants containing S-layer proteins were transferred to a fresh tube and neutralised to pH 7.0 with Tris-HCl.

**Glycoprotein detection**

Glycosylated proteins were detected using Pierce™ Glycoprotein Staining Kit according to the manufacturer’s instructions (Detail in Supplementary Materials). Briefly, S-layer preparations were separated on a 12% Novex NuPAGE Bis-Tris SDS-PAGE gel (Life Technologies), following which the gel was fixed with 100 ml 50% methanol for 30 min. The gel was washed twice in 100 ml 3% acetic acid for 10 min before transferring to 25 ml Oxidising Solution for 15 min. The gel was washed three times in 100 ml 3% acetic acid for 5 min before addition of 25 ml Glycoprotein Staining Reagent for 15 min. The gel was transferred to 25 ml Reducing Solution for 5 min before final extensive washing in 3% acetic acid and ultra-pure water.

**Supplementary results**

**Subgroup analysis of community-onset CDI and hospital-onset CDI**

The increase in severe CDI with more dehydration and/or hypoalbuminemia and bloody diarrhea in patients with RT023-CDI that was observed, could be explained by the increased onset of symptoms in the community for CDI due to RT023 and late recognition of CDI. These symptoms of severe CDI could be a reason for admission in these patients (only hospitalized patients are included in this study). Since these admitted patients are from the community, they might have less comorbidity and this may explain the absence of an increase in complicated course or mortality. Other ribotype groups of *C. difficile* contain more patients with onset of symptoms in the hospital who have been admitted because of other diseases than CDI with possibly less severe symptoms of CDI. To correct for this, we compared RT023 with other non-hypervirulent ribotypes and hypervirulent ribotypes on severity of disease separately for hospital onset of symptoms (HO-CDI) and community onset of symptoms (CO-CDI), using the Pearson’s Chi square test or Fisher’s exact test for expected frequencies <5 (Supplementary Table S1).

When compared to other non-hypervirulent ribotypes, RT023 still has increased severe CDI in the CO-CDI group (p=0.019, RT023: 46%(34-57) vs others: 32%(30-35)), but this is not significant in the HO-CDI group (p=0.112, RT023: 22%(10-33) vs others: 14%(12-15)), although this was based on low numbers. The result was unchanged after correcting for age and gender (multivariate logistic regression). However for HO-CDI, diarrhea with dehydration and/or hypoalbuminemia (p=0.038, RT023: 14%(4-23) vs others: 6%(5-7)) and pseudomembranous colitis (p=0.039, RT023: 8%(2-19) vs others:2%(2-3)) were significantly increased in the RT023 group. Bloody diarrhea (p=0.625) and fever and leukocytosis (p=0.520) were not significantly different. For CO-CDI, all subvariables of severe CDI are not significantly different, but apparently they are significant when combined together.

The same occurred when comparing RT023 with RT014/020/295 (for severe CDI: p=0.122 for HO-CDI and p=0.012 for CO-CDI), except for a non-significant difference in diarrhea with dehydration and hypoalbuminemia in HO-CDI analysis (p=0.080, RT023: 14%(4-23) vs RT014/020/295: 7%(4-9)) and a significant increase of bloody diarrhoea in the RT023 group in the CO-CDI analysis (p=0.015, RT023: 14%(6-22) vs RT014/020/295: 6%(3-8)).

When compared to RT001, RT023 still has increased severe CDI in the HO-CDI group (p=0.008, RT023: 22%(10-33) vs RT001: 10%(7-12)), with a significant increase in diarrhea with dehydration and hypoalbuminemia (p=0.002, RT023: 14%(4-23) vs RT001: 3%(1-4)), but no significant effect in the CO-CDI group (p=0.077, RT023: 46%(34-57) vs RT001: 33%(26-41)), including the subvariables. The results for severity of CDI, including its subvariables, remained the same for comparison between RT023 and RT027 or RT078/126, except for no increased bloody diarrhoea in the RT023 group compared to RT078/126 group in the HO-CDI analysis.

In summary, in the CO-CDI group the severity of CDI due to RT023 was significantly increased compared to other non-hypervirulent ribotypes. Patients with HO-CDI don’t have more severe CDI due to RT 023 compared to other non-hypervirulent ribotypes. However, diarrhea with dehydration and/or hypoalbuminemia and/or pseudomembranous colitis were still significantly increased in the RT023 group.

**MLST analysis of clade 3 strains reveals ambiguities**

MLST were identified *in silico* from *de novo* assemblies*.* The six published strains matched their published MLST with the new strains composed of 68 ST005, 10 ST022, and one novel ST (strain OUS23024) (Figure 1). ST005 and ST022 only differ by eight SNPs (seven synonymous and one non-synonymous) in one MLST locus (*atpA*, an ATP synthase). Furthermore, all clade 3 MLST thus reported (ST005, ST022, ST025, ST096(9) and ST285) have a MLST that includes an identical *dxr* gene (allele 4). In contrast strain OUS23024, clustering with ST005 strains, match ST005 at six loci with only a different *dxr* gene (allele 2 compared to allele 4) with nine *dxr* SNPs. The *dxr* allele 2 has, until now, been exclusively found in clade 1.

Two of the ST005 strains matched exactly at six out of seven MLST loci in the assemblies. The aligned reads were used to determine that these two strains matched the ST005 *recA* allele (allele 2). New MLST strain OUS23024 contains a *dxr* allele 2, common to clade 1, which differs from the clade 3 allele 4 by nine SNPs, of which four are synonymous (V20V, Q45Q, G229G, S321S) and five are nonsynonymous (E23D, H24N, V188A, A252T, I279M). OUS23024 clusters with ST022 strains, with the phylogeny of clade 3 strains otherwise cleanly split between ST005 and ST022 (Figure 1A). The six additional samples were included in the phylogeny, with the ST005 and ST022 samples clustering appropriately and the non ST005/022 strains in the outlier group. All outlier strains were ST005 strains (Figure 1A). The two outlier strains (91, 108698) are not RT023 as repeat PCR ribotyping, performed by the London CDRN, demonstrates (Supplementary Figure S3). This may explain in part the divergence from the other clade 3 strains, the majority of which are RT023. Of the 19k SNPs, one third had non-synonymous genetic changes (6245, 32.4%) and 2575 (13.4%) fell outside of coding regions. The SNP rate including the two outliers is one SNP locus per 222.0 bp.

**Toxin analysis of clade 3 strains**

The pathogenicity locus (PaLoc) is a 27 kbp region (*tcdRBEAC*) containing only 75 mutations; 47 (62.6%) are found in the two outlier strains (91 and 108698). Excluding the two outlier strains, the SNP loci rate is one SNP per 978.0 bp in the PaLoc region, 2.5 times less than the SNP rate over the whole genome (one SNP locus per 382.3 bp). In addition, the binary toxin CDT has only one non-synonymous SNP in *cdt* in three strains. These results are presented in full in Supplementary Table S4.

**SNP analysis of trehalose cluster**

Between clade 3 strains there are only a small number of SNPs in the trehalose gene cluster, predominantly in strain 91. This contains SNPs in *treA2* (K297E), *treX* (D270N, W477*) and *ptsT* (P190S). Strain 108698 also has a SNP within *treA2* (A317S). There were no observed SNPs for any of the 80 strains in *treR2*.

**Clade 3 have defective esculinase (beta-glucosidase) gene clusters**

RT023 strains do not form distinctive black colonies on esculin detection agar (10, 11). Esculin is hydrolysed by BglA or BglB, phospho-β-glucosidases, part of the glycosyl transferase family 1 (12). Five *bgl* gene clusters were identified in *C. difficile* strain 630 (RT012, clade 1) (Supplementary Figure S4). Putative esculinase clusters observed in the strain 630 genome include known *bgl* genes *bglA*, *bglG* and *bglF*. Bgl clusters 2 and 4 have an alternative β-glucosidase, *ascB1*, while cluster 3 has an orthologue of *bglA2*, a β-glucosidase with an alternative substrate to esculin (13). Strains R20291 (clade 2), CF5 (clade 4) and M120 (clade 5) form black colonies on esculin agar (10). Bgl clusters 1-4 were observed in all four genomes (Supplementary Figure S4). Bgl cluster 5 was not observed in clade 2, and therefore is unlikely to be important for the esculin hydrolysis phenotype. Analysis of the CD305 genome reveals conservation of clusters 3 and 5, confirming a lack of importance in esculin hydrolysis. Bgl cluster 1 contains a stop codon in *bglA* separating this enzyme into two distinct gene products, cluster 2 is absent, and cluster 4 shows only 55% sequence identity to the strain 630 cluster, with transporter and glucosidase genes replaced with two mannosylglycerate hydrolases (*mh*) and a glycerate kinase (*garK*) (Supplementary Figure S4). Bgl cluster 2 is downstream of the trehalose metabolism *treR-treA* cluster absent from clade 3 strains, showing a large region of carbohydrate transport systems is absent from clade 3 strains. The absence of cluster 2, and the alternate forms of cluster 1 and 4 are consistent between all strains of clade 3 analysed. Representative strains from RT056, RT058 and RT076, which are also negative on esculin agar, retain the cluster 1 stop codon in *bglA*. RT056 contains cluster 2, while RT058 has the Clade 2 and 4 genotype, and RT076 only retain *bglG*. Cluster 3 is present in all these strains and Cluster 4 is divergent from the Clade 3 genotype. We can therefore assume that the stop codon in *bglA* in Cluster 1 is the determinant for an esculinase negative phenotype.

**Appendix S2 Supplementary Figures**

**Supplementary Figure S1: Phylogeny with Isolation Year and Country of Origin**

**
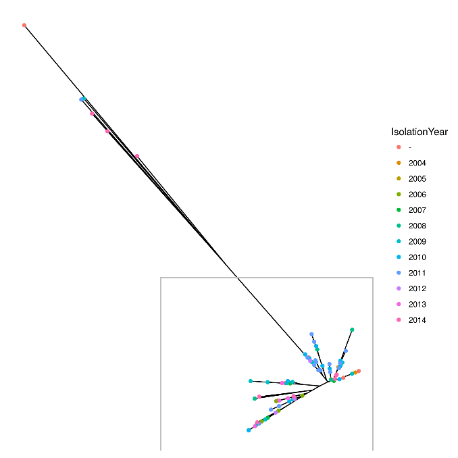

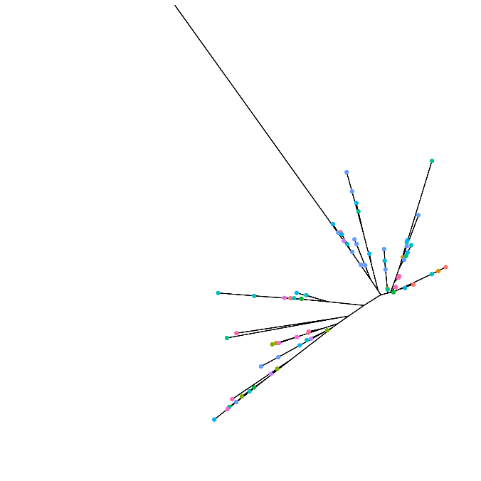
**

**
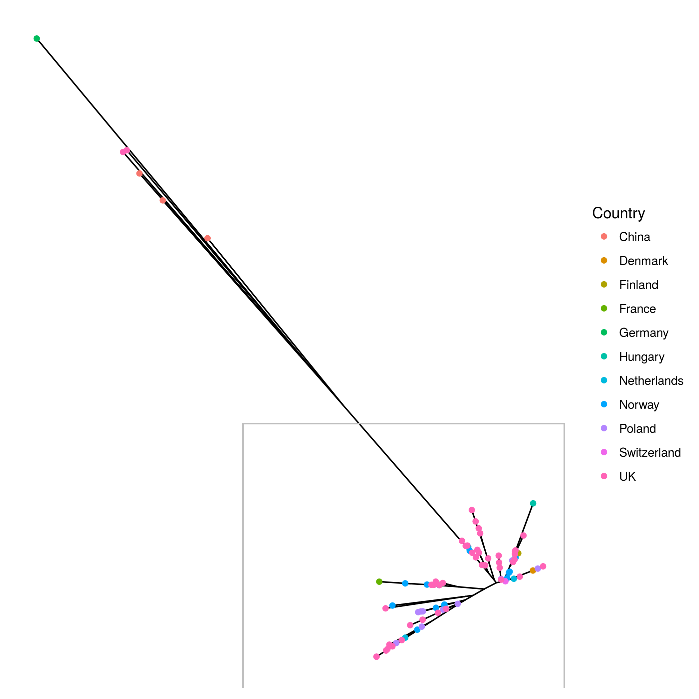

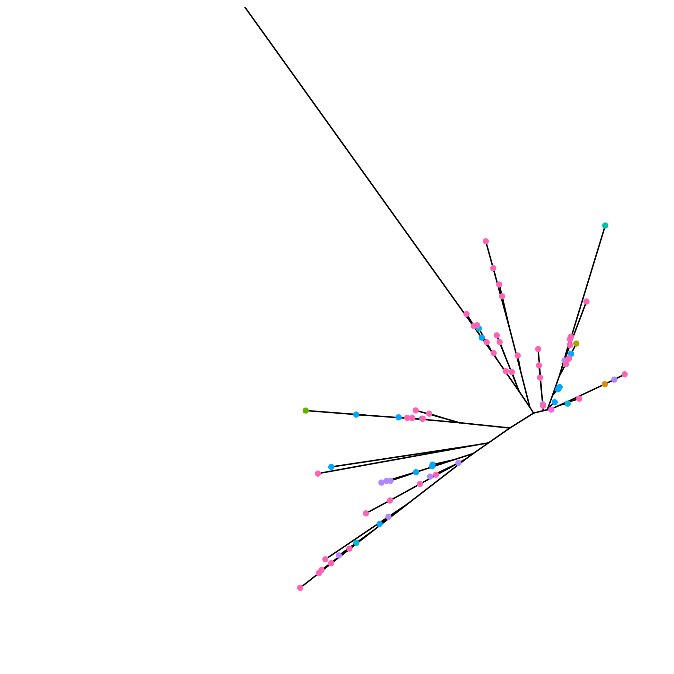
**

**
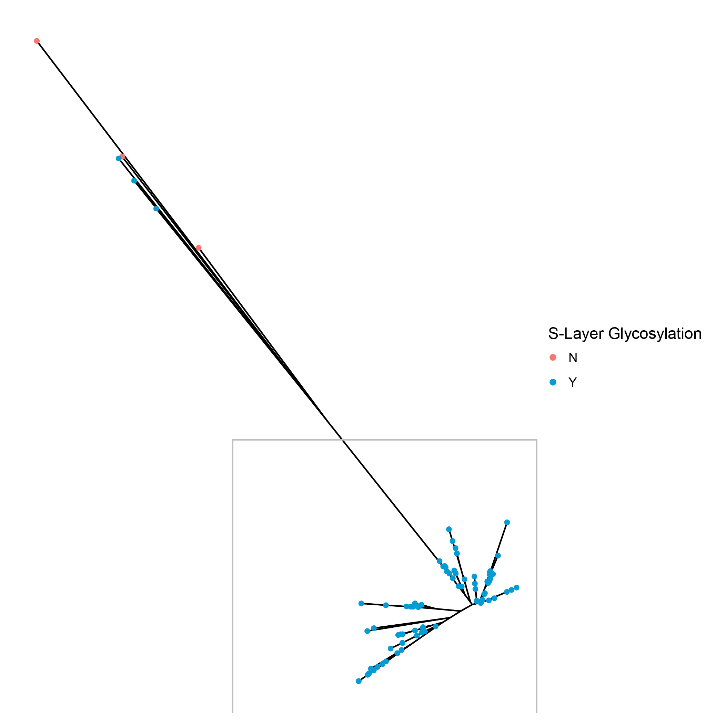
**

**Supplementary Figure S2: Phylogeny with S-layer Glycosylation insertion**


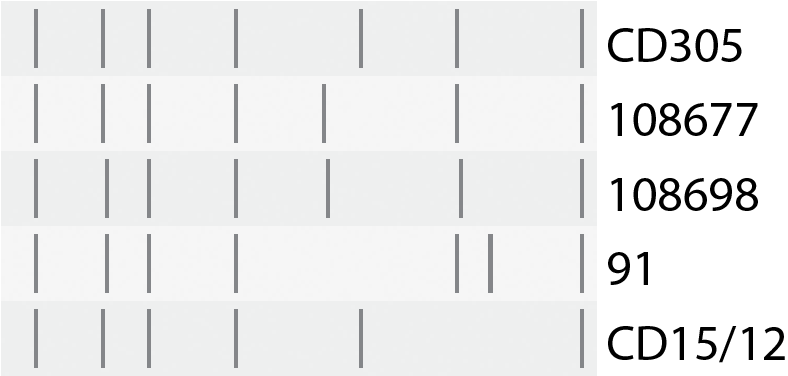


**Supplementary Figure S3: RT analysis of clade 3 strains**

Ribotype (RT) PCR analysis of five clade 3 strains shows a variety of ribotypes. CD305, a known RT023 strain, is compared with variant strains from clade 3. 108677 and 108698 could be misinterpreted as RT023, while 91 and CD15/12 have distinctly different PCR profiles.


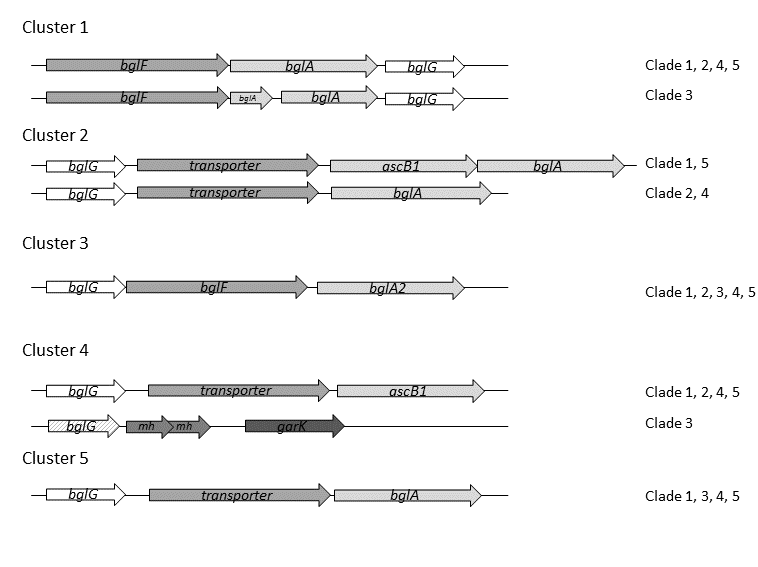


**Supplementary Figure S4: β-glucosidase genes of *C. difficile* show variation between clades**

Schematic demonstrating the five clusters of *bgl* genes observed in *C. difficile*, consisting of *bglA* and *ascB1* β-glucosidase genes, *bglG* a positive regulator, and *bglF* a sensor and transporter. *Mh*, a mannosylglycerate hydrolase, and *garK,* a glycerate kinase, were also observed. Clades where each Bgl cluster is present are indicated.

**Supplementary Table S1. Severity of CDI by RT023 and by other RTs for HO-CDI and CO-CDI**

|  | **RT** |  | **N** | **%** | **Lower CI (95%)** | **Upper CI (95%)** | **p** |
| --- | --- | --- | --- | --- | --- | --- | --- |
| HO-CDI | 023 |  | 11 | 22% | 10% | 33% |  |
|  | Others |  | 313 | 14% | 12% | 15% | 0.112 |
|  | 078/126 |  | 76 | 20% | 16% | 24% | 0.745 |
|  | 027 |  | 13 | 20% | 10% | 30% | 0.869 |
|  | 014/020/295 |  | 67 | 14% | 11% | 17% | 0.122 |
|  | 001 |  | 42 | 10% | 7% | 12% | 0.008 |
| CO-CDI | 023 |  | 32 | 46% | 34% | 57% |  |
|  | Others |  | 477 | 32% | 30% | 35% | 0.019 |
|  | 078/126 |  | 94 | 36% | 31% | 42% | 0.157 |
|  | 027 |  | 14 | 45% | 28% | 63% | 0.959 |
|  | 014/020/295 |  | 102 | 30% | 25% | 35% | 0.012 |
|  | 001 |  | 50 | 33% | 26% | 41% | 0.077 |

HO-CDI: hospital onset of symptoms, CO-CDI community onset of symptoms, RT: ribotype, CI: 95% confidence interval, p: p-value of comparison between RT023 and another group.

**References**

1. Twelfth Annual Report of the National Reference Laboratory for Clostridium difficile and results of the sentinel surveillance May 2017 - May 2018. Available from: <https://www.rivm.nl/sites/default/files/2018-11/Annual%20report%20may%202017-may%202018.pdf>.

2. Tsai IJ, Otto TD, Berriman M. Improving draft assemblies by iterative mapping and assembly of short reads to eliminate gaps. Genome biology. 2010;11(4):R41.

3. Chain PS, Grafham DV, Fulton RS, Fitzgerald MG, Hostetler J, Muzny D, et al. Genomics. Genome project standards in a new era of sequencing. Science (New York, NY). 2009;326(5950):236-7.

4. Bolger AM, Lohse M, Usadel B. Trimmomatic: a flexible trimmer for Illumina sequence data. Bioinformatics (Oxford, England). 2014;30(15):2114-20.

5. Li H, Durbin R. Fast and accurate short read alignment with Burrows-Wheeler transform. Bioinformatics (Oxford, England). 2009;25(14):1754-60.

6. Li H, Handsaker B, Wysoker A, Fennell T, Ruan J, Homer N, et al. The Sequence Alignment/Map format and SAMtools. Bioinformatics (Oxford, England). 2009;25(16):2078-9.

7. Danecek P, Auton A, Abecasis G, Albers CA, Banks E, DePristo MA, et al. The variant call format and VCFtools. Bioinformatics (Oxford, England). 2011;27(15):2156-8.

8. Zerbino DR. Using the Velvet de novo assembler for short-read sequencing technologies. Current protocols in bioinformatics. 2010;Chapter 11:Unit 11.5.

9. Stabler RA, Dawson LF, Valiente E, Cairns MD, Martin MJ, Donahue EH, et al. Macro and micro diversity of Clostridium difficile isolates from diverse sources and geographical locations. PloS one. 2012;7(3):e31559.

10. Connor MC, Fairley DJ, McKenna JP, Marks NJ, McGrath JW. Clostridium difficile Ribotype 023 lacks the ability to hydrolyse esculin, leading to false negative results on chromogenic agar. Journal of clinical microbiology. 2016.

11. Reigadas E, Alcala L, Marin M, Martin A, Bouza E. C. difficile PCR-ribotype 023 might go undetected when using ChromId C. difficile agar. Anaerobe. 2017;44:34-5.

12. Old LA, Lowes S, Russell RR. Genomic variation in Streptococcus mutans: deletions affecting the multiple pathways of beta-glucoside metabolism. Oral microbiology and immunology. 2006;21(1):21-7.

13. Yu WL, Jiang YL, Pikis A, Cheng W, Bai XH, Ren YM, et al. Structural insights into the substrate specificity of a 6-phospho-beta-glucosidase BglA-2 from Streptococcus pneumoniae TIGR4. The Journal of biological chemistry. 2013;288(21):14949-58.
